# What Is Ontic and What Is Epistemic in the Quantum Mechanics of Spin?

**DOI:** 10.3390/e27030315

**Published:** 2025-03-18

**Authors:** Ariel Caticha

**Affiliations:** Physics Department, University at Albany-SUNY, Albany, NY 12222, USA; acaticha@albany.edu

**Keywords:** foundations of quantum mechanics, entropic dynamics, Hamilton–Killing flows, spin

## Abstract

Entropic Dynamics (ED) provides a framework that allows the reconstruction of the formalism of quantum mechanics by insisting on ontological and epistemic clarity and adopting entropic methods and information geometry. Our present goal is to extend the ED framework to account for spin. The result is a *realist* ψ*-epistemic model* in which the ontology consists of a particle described by a definite position plus a discrete variable that describes Pauli’s peculiar two-valuedness. The resulting dynamics of probabilities is, as might be expected, described by the Pauli equation. What may be unexpected is that the generators of transformations—Hamiltonians and angular momenta, including spin, are all granted clear epistemic status. To the old question, ‘what is spinning?’ ED provides a crisp answer: *nothing is spinning*.

## 1. Introduction

The framework of Entropic Dynamics (ED) allows the formulation of dynamical theories as an application of the method of maximum entropy [1,2,3]. It allows, among other things, a derivation of the Schrödinger equation, including its linear and complex structure, and it also clarifies the most controversial aspect of quantum mechanics (QM)—its interpretation [3,4,5]. The controversy centers around the question of what is real or *ontic* at the microscopic quantum level. Furthermore, it is not clear either how the *epistemic* aspects of the theory are handled. Are probabilities already present at the microscopic level, or do they only arise at the macroscopic classical level when measurements are performed? Could we perhaps need some altogether different type of quantum or exotic probability?

In the ED approach, the main concern is to achieve ontological and epistemological clarity and, therefore, before any further discussion, it may be desirable to be explicit about the terminology: The paradigmatic ontic concept is matter; a quantity is said to be ‘ontic’ when it refers to something real, substantial. The paradigmatic epistemic quantities are probabilities and wave functions; a concept is ‘epistemic’ when it refers to the state of belief, opinion, or knowledge of an agent (who, for our current purposes, we shall assume to be an ideally rational agent). Models such as ED that invoke ontic variables (e.g., position) while the wave function remains fully epistemic are often described as “realist ψ-epistemic models.” There exist powerful no-go theorems that rule out large families of such models—the so-called “ontological” models—because they disagree with QM. For an extended list of references and a discussion of how ED evades those no-go theorems, see [6]. Briefly, ED is realist and ψ-epistemic, but it is not an ontological model.

On a related issue, it is important to emphasize that the distinction between ontic and epistemic is not the same as the distinction between objective and subjective. Probabilities, for example, are always fully epistemic (because they codify credences or degrees of belief) but they can lie anywhere in the spectrum from objective to subjective. To be explicit, probabilities in QM are fully objective, but in other contexts, probabilities can be subjective because two agents could hold different beliefs as a result of different priors or different data. This paper, however, is not about philosophy; it is about extending the ED framework to the discussion of the quantum mechanics of spin.

Previous work on ED dealt with *N* spinless particles. A central assumption is that the particles have definite positions, which represents a major departure from the standard interpretation of quantum mechanics, according to which definite values can be attained but only as the result of a measurement. We should be explicit: QM lives in 3N-dimensional configuration space, and, as far as positions are concerned, ED agrees with Einstein’s view that spatially separated objects have definite separate ontic states. In particular, in the ED description of the double slit experiment for a single particle, we might not know which slit the particle goes through, but the particle definitely goes through one slit or the other. In this paper, we deal with a single particle with spin; in a future paper, we shall extend the framework to several identical particles with spin.

In 1923, Pauli introduced a “peculiar, classically not describable two-valuedness” [7,8] associated with spin and to the exclusion principle. Spin is angular momentum, and that, in itself, is not particularly strange, but ever since Uhlenbeck and Goudsmit’s 1925 idea of spin [9], the nature of what, if anything, is actually spinning, and the nature of the peculiar two-valuedness has been and continues to be a subject of interest and numerous studies. The standard Copenhagen interpretation forgoes the possibility of visualizable spin models. In other interpretations, however, spin is an ontic variable that is variously attributed either to a spinning particle or to the helical motion of a point particle as guided by a wave function, and the latter might be a Bohmian wave function, a Pauli spinor, a Dirac spinor, or a real spinor function in the language of geometric algebra. A non-exhaustive list of references includes [10,11,12,13,14,15,16,17,18,19,20,21,22,23]; in particular, refs. [14,16] deal with the interpretation of relativistic spin and the Dirac equation, and [21,22,23] contain a wealth of recent references, both to foundational and more computational applications. None of these models, however, provide insights as to why the Pauli equation takes the particular form it does (e.g., how does one derive the linearity, the adoption of complex numbers, and so on).

In a previous work [24,25], we presented a nonrelativistic ED model for a single spin-1/2 point particle. The position of the particle was assumed to be the *only ontic variable* and spin was recovered as a *property of its epistemic wave function*. The model was successful in the sense that it provided a reconstruction of the single-particle Pauli equation but two features have hindered its satisfactory extension to several particles. The first is that the four real degrees of freedom of the single-particle spinor allow an elegant geometric interpretation in terms of a probability density plus the three Euler angles that define the rotation from a fixed lab frame to a spatially varying “spin” frame attached to the particle [24]. Unfortunately, this very appealing feature does not generalize to several particles because it is not, in general, possible to attach a separately rotating spin frame to each particle. One specific goal of the present paper is to provide an ED reconstruction of the Pauli equation that could, in principle, be extended to several particles because it does not rely on individual spin frames.

The second feature is relevant to the eventual extension to identical particles. The purpose of deploying an information-based framework such as ED to reconstruct QM is to provide natural explanations for the typical quantum effects—interference, entanglement, tunneling, etc. The Pauli exclusion principle, however, has so far proved resistant in the sense that it could only be implemented by force, i.e., by an ad hoc, unexplained antisymmetrization of the wave function. Here, we take a first step towards a more natural explanation.

It turns out that, among all quantum effects, the exclusion principle is unique in that it is remarkably robust. While effects such as interference, entanglement, tunneling, etc., are all destroyed by noise, the exclusion principle can survive under the most extreme conditions, such as, for example, in the interior of stars. A natural explanation would follow from the observation that quantum effects that are sensitive to noise and decoherence can all be traced to the wave function, i.e., to the epistemic sector of ED. The robustness of the exclusion principle strongly suggests that its explanation lies in the ontic sector.

We shall reconstruct the nonrelativistic one-particle Pauli equation by enlarging the ontology to include both the position of the point particle and Pauli’s discrete two-valued variable. This answers the question ‘what is real?’ and defines what variables we are uncertain about (Section 2). The probabilities of these variables form a statistical manifold—the epistemic configuration space—and its associated cotangent bundle constitutes the epistemic phase space. Next, in Section 3, we briefly discuss the kinematics of Hamilton–Killing flows, which singles out those special curves that are adapted to the natural geometrical structures of the epistemic phase space. The discussion of which among those special curves qualify to describe evolution in time—this is the actual entropic dynamics—starts in Section 4 where we study the ED of infinitesimally short steps, followed by the construction of an entropic notion of time in Section 5, and the derivation of a continuity equation for the evolution of probabilities in Section 6. In Section 7, we derive the corresponding Hamiltonian and the Pauli equation. The reconstruction of orbital and spin angular momenta as generators of those Hamilton–Killing flows that also describe rotations is given in Section 8. Some final thoughts and conclusions are collected in Section 9. To make this paper somewhat self-contained, some material presented in [4] is reproduced here. However, the present paper reflects substantial differences: it derives subquantum trajectories that are non-differentiable and Brownian, while in [4], the trajectories are smooth and Bohmian; furthermore, the addition of a discrete two-valued variable requires a revised treatment of time.

## 2. The Ontic and the Epistemic Sectors

The first step is to specify the subject matter—the ontology. We consider a point particle living in a flat Euclidean space. The particle is assumed to have a *definite* position described as $x=\{x^{a},\;a=1,\;2,\;3\}$ in Cartesian coordinates. In addition, we assume the particle occupies a definite state denoted k={−1,+1}, which corresponds to Pauli’s peculiar, “classically not describable two-valuedness”. Both the assumption of a definite *x* and of a definite *k* already represent a major departure from the standard Copenhagen interpretation.

Next, we discuss the epistemic sector. ED is a dynamics of probabilities. The goal is to study the evolution of the joint probability distribution ρ(k,x) and its canonically conjugate momentum ξ(k,x). We adopt the following notation: we shall often abbreviate k=±1 by k=± and write

(1) $$\rho \left(k,x\right)=\rho_{kx}=\rho_{\pm x}\;\mathrm{and}\;\xi \left(k,x\right)=\xi_{kx}=\xi_{\pm x}.$$

As discussed in [3,4,5] it is convenient to transform from the generalized coordinates (ρ,ξ) to complex coordinates, known as the wave function,

(2) Ψ(x)=ψ+xψ−xwhereψ±x=ψkx=ρkx1/2expiℏξkx.

Clearly, the wave function Ψ(x) also belongs in the epistemic sector. The new canonically conjugate pairs are $\left(\psi_{kx},i\hbar \psi_{kx}^{*}\right)$ and the transformation $\left(\rho_{kx},\xi_{kx}\right)\to \left(\psi_{kx},i\hbar \psi_{kx}^{*}\right)$ is a canonical transformation. The generators of translations and rotations, the momentum $\tilde{P}\left[\rho ,\xi \right]$ and the angular momentum $\tilde{J}\left[\rho ,\xi \right]$, are quantities that obviously also belong in the epistemic sector.

The weight of tradition leads us to refer to *k* as ‘spin’, but this might not be fully appropriate because *k* is ontic while spin, being an angular momentum, is an epistemic quantity. We might also refer to *k* as a ‘qubit’, but some caution, however, is called for because of the need to distinguish an ontic qubit (a discrete two-valued ontic variable like *k*) from an epistemic qubit (a two-dimensional Hilbert space). It seems clear that talking about spin or about qubits without being aware of what is ontic and what is epistemic in QM will lead to considerable confusion. Interestingly, the same kind of confusion can arise with the classical term ‘bit’ which applies both to the ontic bit (which refers to binary subsystem in the physical memory of a digital computer) and to the epistemic bit (which refers to a unit of amount of information as measured by the Shannon entropy). This kind of confusion is often reflected in expressions such as “information is physical”. Nevertheless, tradition weighs heavy, and we shall often use the terms ‘spin’ or ‘qubit’ and trust that whether one refers to the ontic or the epistemic version can be understood from the context.

## 3. Kinematics: Hamilton–Killing Flows

The discussion of Hamilton–Killing or HK flows can be carried out by a straightforward extension of the treatment for discrete variables (e.g., the quantum die) in [5] and for continuous variables (particle positions) in [4]. Here, we shall omit most technical details; for a pedagogical discussion, see [3].

Once local coordinates $\left\{\rho_{kx},\xi_{kx}\right\}$ on the epistemic phase space (or e-phase space) have been established, there is a natural choice of symplectic form,

(3) $$\Omega \overset{\mathrm{def}}{=}\int dx\sum_{k}\left(\;\tilde{\nabla }\rho_{kx}⊗\tilde{\nabla }\xi_{kx}-\tilde{\nabla }\xi_{kx}⊗\tilde{\nabla }\rho_{kx}\right),$$

where $\;\tilde{\nabla }$ is the gradient in e-phase space. Alternatively, we can do a canonical transformation to complex coordinates $\left\{\psi_{kx},i\hbar \psi_{kx}^{*}\right\}$, Equation (2), and let the coordinates of a point Ψ in e-phase space be

(4) $$\Psi^{\mu x}=\left(\Psi^{1x},\Psi^{2x},\Psi^{3x},\Psi^{4x}\right)=\left(\psi_{+x},i\hbar \psi_{+x}^{*},\psi_{-x},i\hbar \psi_{-x}^{*}\right),$$

then

(5) $$\Omega \overset{\mathrm{def}}{=}\int dx\sum_{k}\left(\;\tilde{\nabla }\psi_{kx}⊗\tilde{\nabla }i\hbar \psi_{kx}^{*}-\tilde{\nabla }i\hbar \psi_{kx}^{*}⊗\tilde{\nabla }\psi_{kx}\right),$$

and the tensor components of Ω are

(6) $$\left[\Omega_{\mu x,\mu^{\prime }x^{\prime }}\right]=\left[\begin{array}{cccc} 0 & 1 & 0 & 0\\ -1 & 0 & 0 & 0\\ 0 & 0 & 0 & 1\\ 0 & 0 & -1 & 0 \end{array}\right]\;\delta \left(x,x^{\prime }\right).$$

(Notation: $\left[\Omega_{\mu x,\mu^{\prime }x^{\prime }}\right]$ is a 4×4 matrix the elements of which, $\Omega_{\mu \mu^{\prime }}$, are functions of *x* and $x^{\prime }$).

Consider a curve $\Psi^{\mu x}\left(\tau \right)$ on the e-phase space parametrized by τ and let

(7) $$\bar{H}=\frac{d}{d\tau }=H^{\mu x}\frac{\delta }{\delta \Psi^{\mu x}}\;\mathrm{with}\;H^{\mu x}\left[\Psi \right]=\frac{d\Psi^{\mu x}}{d\tau }$$

be the tangent vector at Ψ. We are interested in those special curves that are naturally adapted to the symplectic geometry in the sense that they preserve Ω, i.e.,

(8) $${£}_{\bar{H}}\Omega =0,$$

where ${£}_{\bar{H}}$ is the Lie derivative along $\bar{H}\left[\Psi \right]$ (this is a directional derivative on a curved space). By Poincare’s lemma, requiring ${£}_{\bar{H}}\Omega =0$ (a vanishing “curl”) implies that the covector $\Omega_{\mu x,\mu^{\prime }x^{\prime }}H^{\mu^{\prime }x^{\prime }}$ is the gradient of a scalar function [26], denoted $\tilde{H}\left[\Psi \right]$,

(9) $$\Omega_{\mu x,\mu^{\prime }x^{\prime }}H^{\mu^{\prime }x^{\prime }}=\frac{\delta }{\delta \Psi^{\mu x}}\tilde{H}\left[\Psi \right].$$

($\tilde{H}\left[\Psi \right]$ is the scalar function associated with the vector $\bar{H}\left[\Psi \right]$). Substituting (6) and (7), this is rewritten as

(10) $$\frac{d\psi_{kx}}{d\tau }=\frac{\delta \tilde{H}}{\delta (i\hbar \psi_{kx}^{*})}\;\mathrm{and}\;\frac{d(i\hbar \psi_{kx}^{*})}{d\tau }=-\frac{\delta \tilde{H}}{\delta \psi_{kx}},$$

which are recognized as Hamilton’s equations for a Hamiltonian function $\tilde{H}$. This is the reason for Hamiltonians in physics: the congruence of curves that preserve the natural symplectic geometry, ${£}_{\bar{H}}\Omega =0$ are called *Hamilton flows*. They are generated by *Hamiltonian vector fields* $\bar{H}$ or, equivalently, by their associated *Hamiltonian functions* $\tilde{H}$. Our challenge, to be addressed next, is to find Hamiltonians $\tilde{H}$ that yield interesting flows.

It turns out that, in addition to the symplectic geometry Ω, the e-phase also has a natural metric geometry inherited from the information geometry of the epistemic configuration space, which is a statistical manifold. A straightforward extension from [3,4,5] yields a particularly simple line element,

(11) $$\delta \ell^{2}=\int dxdx^{\prime }\sum_{\mu \mu^{\prime }}G_{\mu x,\mu^{\prime }x^{\prime }}\delta \Psi^{\mu x}\delta \Psi^{\mu^{\prime }x^{\prime }}=2\hbar \int dx\sum_{k}\;\delta \psi_{kx}\delta \psi_{kx}^{*}.$$

The components of the metric tensor *G* and its inverse are

(12) $$\left[G_{\mu x,\mu^{\prime }x^{\prime }}\right]=-i\left[\begin{array}{cccc} 0 & 1 & 0 & 0\\ 1 & 0 & 0 & 0\\ 0 & 0 & 0 & 1\\ 0 & 0 & 1 & 0 \end{array}\right]\;\delta \left(x,x^{\prime }\right),\;\left[G^{\mu x,\mu^{\prime }x^{\prime }}\right]=i\left[\begin{array}{cccc} 0 & 1 & 0 & 0\\ 1 & 0 & 0 & 0\\ 0 & 0 & 0 & 1\\ 0 & 0 & 1 & 0 \end{array}\right]\;\delta \left(x,x^{\prime }\right).$$

A remarkable further development is that the contraction of the symplectic form Ω, Equation (6), with the inverse metric $G^{-1}$ allows us to construct a tensor *J* with components

(13) $${J^{\mu x}}_{\mu^{\prime }x^{\prime }}=-\int dx^{\prime \prime }\sum_{\mu^{\prime \prime }}G^{\mu x,\mu^{\prime \prime }x^{\prime \prime }}\Omega_{\mu^{\prime \prime }x^{\prime \prime },\mu x^{\prime }}\;\mathrm{and}\;\left[{J^{\mu x}}_{\mu^{\prime }x^{\prime }}\right]=\left[\begin{array}{cccc} i & 0 & 0 & 0\\ 0 & -i & 0 & 0\\ 0 & 0 & i & 0\\ 0 & 0 & 0 & -i \end{array}\right]\;\delta \left(x,x^{\prime }\right).$$

What makes the tensor *J* special is that its square is

(14) $$\int dx^{\prime \prime }\sum_{\mu^{\prime \prime }}{J^{\mu x}}_{\mu^{\prime \prime }x^{\prime \prime }}{J^{\mu^{\prime \prime }x^{\prime \prime }}}_{\mu^{\prime }x^{\prime }}=-{\delta^{\mu }}_{\mu^{\prime }}\delta \left(x,x^{\prime }\right).$$

In other words, the action of $J^{2}$ is equivalent to multiplying by −1, which means that *J* provides a complex structure. This is the reason for complex numbers in QM and explains why it was convenient to introduce wave functions (i.e., complex coordinates) in the first place.

Next, we take advantage of the metric geometry and seek those doubly special curves that preserve both the symplectic and the metric geometries,

(15) $${£}_{\bar{H}}\Omega =0\;\mathrm{and}\;{£}_{\bar{H}}G=0.$$

We want $\bar{H}$ to be both a Hamilton and a Killing vector field; then the associated Hamiltonian function $\tilde{H}$ will generate Hamilton–Killing flows. Imposing further that the HK flows preserve the normalization of probabilities restricts the Hamiltonian function to those that are bilinear in $\psi_{kx}$ and $\psi_{kx}^{*}$,

(16) $$\tilde{H}=\int dxdx^{\prime }\sum_{kk^{\prime }}\psi_{kx}^{*}{\hat{H}}_{kx,k^{\prime }x^{\prime }}\psi_{k^{\prime }x^{\prime }}.$$

$\tilde{H}$ is the Hamiltonian that generates evolution on the e-phase space and is, accordingly, called the e-Hamiltonian. From Equation (10), the corresponding equation of evolution is

(17) $$i\hbar \frac{\partial \psi_{kx}}{\partial \tau }=\frac{\delta \tilde{H}}{\delta \psi_{kx}^{*}}=\int dx^{\prime }\sum_{k^{\prime }}{\hat{H}}_{kx,k^{\prime }x^{\prime }}\psi_{k^{\prime }x^{\prime }},$$

which is recognized as a Schrödinger equation. Hamilton-Killing flows explain the linearity of QM.

At this point in the development, τ is just a parameter along a curve; there is no implication that the curve represents time evolution and τ is time or that the curve is generated by rotations about an axis and τ is a rotation angle. To identify those special curves demands that additional information be incorporated into the analysis in order to constrain the e-Hamiltonian function $\tilde{H}$ beyond the generic bilinear form, Equation (16).

## 4. The Entropic Dynamics of Short Steps

Beyond the reconstruction of the framework of QM, including its interpretation, another central goal of ED is the formulation of an information-based notion of time. This involves the introduction of the concept of an instant, the notion that the instants are suitably ordered, and a convenient definition of duration. Remarkably, by its very construction associated with entropic dynamics, there is a natural arrow of entropic time.

The physically relevant information that will allow us to recover a satisfactory concept of time is that the particle follows a continuous trajectory in space. The continuity allows the dynamics to be analyzed as a sequence of a large number of infinitesimally short steps $kx\to k^{\prime }x^{\prime }$. The difficulty in the presence of spin is that the term ‘short’ refers to a short *spatial* step,

(18) $$\Delta x^{a}=x^{\prime a}-x^{a}\to 0,$$

but the Δk steps could be discontinuous, either Δk=0 or ±2. Thus, in order to incorporate the physical information that trajectories are continuous, the *k* variables must, at least provisionally, be removed. This is achieved by averaging over the initial *k* and the final $k^{\prime }$.

The evolution of the joint probability $\rho_{kx}$ is given by

(19) $$\rho_{k^{\prime }x^{\prime }}^{\prime }=\int dx\sum_{k}P\left(k^{\prime }x^{\prime }|kx\right)\rho_{kx}.$$

Averaging over the final $k^{\prime }$ and using the product rule,

(20) $$\rho_{kx}=\rho_{x}\rho_{k|x}\;\mathrm{where}\;\rho_{x}=\sum_{k}\rho_{kx},$$

gives

(21) $$\rho_{x^{\prime }}^{\prime }=\int dx\sum_{kk^{\prime }}P\left(k^{\prime }x^{\prime }|kx\right)\rho_{kx}=\int dx\left[\sum_{kk^{\prime }}P\left(k^{\prime }x^{\prime }|kx\right)\rho_{k|x}\right]\rho_{x}.$$

Therefore, the *spatial probability* $\rho_{x}$ evolves according to

(22) $$\rho_{x^{\prime }}^{\prime }=\int dx\;P\left(x^{\prime }|x\right)\rho_{x}\;\mathrm{where}\;P\left(x^{\prime }|x\right)=\sum_{kk^{\prime }}P\left(k^{\prime }x^{\prime }|kx\right)\rho_{k|x}.$$

Our immediate goal is to derive the spatial transition probability $P(x^{\prime }|x)$. Our argument closely follows the ED of scalar particles. The transition probability $P(x^{\prime }|x)$ is found by maximizing the entropy,

(23) $$S\left[P,Q\right]=-\int dx^{\prime }\;P\left(x^{\prime }|x\right)\log\frac{P(x^{\prime }|x)}{Q(x^{\prime }|x)},$$

relative to a prior $Q(x^{\prime }|x)$ and subject to constraints that implement the physically relevant information that we associate with quantum evolution [1,2,3].

We require the prior $Q(x^{\prime }|x)$ to codify the physical information that all short steps have in common, specifically, that they are infinitesimally short, but *Q* should otherwise remain maximally non-informative; it should not induce a preferred directionality to the motion. Such a prior can itself be derived by maximizing an entropy,

(24) $$S\left[Q,\mu \right]=-\int dx^{\prime }\;Q\left(x^{\prime }|x\right)\log\frac{Q(x^{\prime }|x)}{\mu (x^{\prime }|x)},$$

relative to some sufficiently smooth distribution $\mu (x^{\prime }|x)$ and subject to normalization and the rotationally invariant constraint,

(25) $$\langle \delta_{ab}\Delta x^{a}\Delta x^{b}\rangle =\kappa ,$$

and the small quantity κ is specified below. The result is a Gaussian distribution,

(26) $$Q\left(x^{\prime }|x\right)\propto \exp-\frac{1}{2}\alpha \delta_{ab}\Delta x^{a}\Delta x^{b},$$

where α is a Lagrange multiplier. To enforce short steps, we shall eventually take the limit α→∞, which amounts to taking κ→0. (In the α→∞ limit, the prior $Q(x^{\prime }|x)$ becomes independent of the choice of the distribution $\mu (x^{\prime }|x)$ provided it is sufficiently smooth.)

The physical information about directionality and correlations is introduced via a “phase” constraint that follows the standard ED strategy. For *scalar* particles one introduces a “drift potential” $\varphi_{x}$ that is canonically conjugate to the probability distribution $\rho_{x}$; it obeys the canonical Poisson brackets,

(27) $$\left\{\rho_{x},\varphi_{x^{\prime }}\right\}=\delta_{xx^{\prime }}\;,\left\{\rho_{x},\rho_{x^{\prime }}\right\}=0=\left\{\varphi_{x},\varphi_{x^{\prime }}\right\}.$$

(Eventually, a canonical transformation is performed that replaces $\varphi_{x}$ by a more convenient momentum $\xi_{x}$). Then, the relevant dynamical information is imposed via a constraint on the component of the expected displacement $\langle \Delta x^{a}\rangle$ along the gradient of φ,

(28) $$\langle \Delta x^{a}\rangle \partial_{a}\varphi =\kappa^{\prime }.$$

Here, to account for spin, we introduce “two drift potentials”, $\varphi_{k}\left(x\right)=\varphi_{kx}$, that are canonically conjugate to the distribution $\rho_{kx}$,

(29) $$\left\{\rho_{kx},\varphi_{k^{\prime }x^{\prime }}\right\}=\delta_{kk^{\prime }}\delta_{xx^{\prime }}\;,\left\{\rho_{kx},\rho_{k^{\prime }x^{\prime }}\right\}=0=\left\{\varphi_{kx},\varphi_{k^{\prime }x^{\prime }}\right\}.$$

Since continuity, i.e., short steps, reflects the purely spatial aspect of the transition probability $P(x^{\prime }|x)$, the phase constraint takes the form

(30) $${\langle \Delta x^{a}\rangle }_{x}{\bar{\partial_{a}\varphi }}_{x}=\kappa^{\prime },\;\mathrm{where}\;{\langle \Delta x^{a}\rangle }_{x}=\int dx^{\prime }\;P\left(x^{\prime }|x\right)\;\Delta x^{a},$$

and where the effective gradient $\bar{\partial_{a}\varphi }$ is obtained by averaging the gradients $\partial_{a}\varphi_{kx}$ over *k*,

(31) $${\bar{\partial_{a}\varphi }}_{x}=\sum_{k}\rho_{k|x}\partial_{a}\varphi_{kx},$$

and $\rho_{k|x}=\rho_{kx}/\rho_{x}$. The effect of interactions with an external electromagnetic field is handled in the same way as for scalar particles: the gauge constraint is

(32) $${\langle \Delta x^{a}\rangle }_{x}A_{a}\left(\vec{x}\right)=\kappa^{\prime \prime }.$$

As is usual in applications of the maximum entropy method, the specification of the numerical values of $\kappa^{\prime }$ and $\kappa^{\prime \prime }$ is most conveniently handled indirectly through the corresponding Lagrange multipliers.

Next, we maximize Equation (23) subject to (30), (32), and normalization. The result is

(33) $$P\left(x^{\prime }|x\right)\propto \exp\left\{-\frac{\alpha }{2}\delta_{ab}\Delta x^{a}\Delta x^{b}+\alpha^{\prime }\left({\bar{\partial_{a}\varphi }}_{x}-\beta A_{ax}\right)\Delta x^{a}\right\}$$

where the Lagrange multipliers α, $\alpha^{\prime }$ will be specified shortly, and the multiplier β will eventually be interpreted as the electric charge, β=q/c. Alternatively, we can rewrite $P(x^{\prime }|x)$ as

(34) $$P\left(x^{\prime }|x\right)=\frac{1}{Z}\exp\left[-\frac{\alpha }{2}\;\delta_{ab}\left(\Delta x^{a}-\Delta {\bar{x}}_{x}^{a}\right)\left(\Delta x^{b}-\Delta {\bar{x}}_{x}^{b}\right)\right]$$

where

(35) $$\Delta {\bar{x}}_{x}^{a}=\frac{\alpha^{\prime }}{\alpha }\delta^{ab}\left[{\bar{\partial_{b}\varphi }}_{x}-\beta A_{bx}\right]={\langle \Delta x^{a}\rangle }_{x}$$

is the expected displacement. Using Equation (22) we can check that

(36) $$\begin{array}{cc} \Delta {\bar{x}}_{x}^{a} & =\int dx^{\prime }\;P\left(x^{\prime }|x\right)\;\Delta x^{a}\\ \; & =\sum_{k}\rho_{k|x}\int dx^{\prime }\;\sum_{k^{\prime }}P\left(k^{\prime }x^{\prime }|kx\right)\;\Delta x^{a}=\sum_{k}\rho_{k|x}{\langle \Delta x^{a}\rangle }_{kx} \end{array}$$

is just the spatial displacement averaged over the *k* variable. (For fixed *x* the expectations over *k* and $x^{\prime }$ commute).

From (34), a generic displacement $\Delta x^{a}$ can be expressed as the sum of an expected drift, Equation (35), plus a fluctuation $\Delta w^{a}$,

(37) $$\Delta x^{a}=x^{\prime a}-x^{a}=\Delta {\bar{x}}^{a}+\Delta w^{a}\;\;,$$

where

(38) $${\langle \Delta w^{a}\rangle }_{x}=0\;\mathrm{and}\;{\langle \Delta w^{a}\Delta w^{b}\rangle }_{x}=\frac{1}{\alpha }\delta^{ab}.$$

## 5. Entropic Time: Instants and Duration

In ED, time is introduced as a book-keeping device designed to keep track of the accumulation of short steps ([3,4]). It involves identifying suitable notions of ordered “instants” and of the separation or duration between “successive” instants. Just as the prototype of a classical clock is a free particle that “measures” equal intervals by registering equal displacements, the prototype of a quantum clock is the quantum system itself. This implies that in order to recover a notion of time as a continuous succession of instants, it is necessary to appeal to the continuity of spatial trajectories.

Referring to the discussion in the previous section, specifying the duration or interval $\Delta t=t^{\prime }-t$ between successive instants amounts to specifying the relation between Δt and the multipliers α and $\alpha^{\prime }$ [4]. The basic criterion is convenience: “duration is defined so that motion looks simple”. To reflect the translational symmetry of a nonrelativistic Newtonian space and time, we choose $\alpha^{\prime }$ and α to be independent of *x* and *t* so that time flows “equably everywhere and everywhen”. We *define* Δt so that $\alpha^{\prime }/\alpha \propto \Delta t$. Then, as we see from (35), the particle has a well-defined expected velocity. For later convenience, the proportionality constant is written as 1/m,

(39) $$\frac{\alpha^{\prime }}{\alpha }=\frac{1}{m}\Delta t.$$

At this point, the constant *m* has no special significance, but once we derive the Pauli equation, it will be recognized as the particle’s mass. It remains to specify α. We choose α so that for sufficiently short steps, the expected fluctuations, ${\langle \Delta w^{a}\Delta w^{b}\rangle }_{x}$, increase by equal amounts in equal intervals Δt. Referring to Equation (38) this is achieved by setting

(40) $$\frac{1}{\alpha }=\frac{\eta }{m}\Delta t\;\mathrm{so}\;\mathrm{that}\;\alpha^{\prime }=\frac{1}{\eta }.$$

where a new constant η is introduced. We emphasize that these choices of α and $\alpha^{\prime }$ are not arbitrary as they lead to a natural physical interpretation: duration is defined so it reflects the symmetries of Newtonian space and time, and so that over short steps particles have well-defined expected velocities while equal intervals of entropic time correspond to equal increases of the variance $\langle \Delta w^{2}\rangle$. Indeed, substituting (40) into Equations (35) and (38) we find that a generic displacement is

(41) $$\Delta x^{a}=\Delta {\bar{x}}_{x}^{a}+\Delta w^{a}=b_{x}^{a}\Delta t+\Delta w^{a},$$

where

(42) $$b_{x}^{a}=\frac{{\langle \Delta x^{a}\rangle }_{x}}{\Delta t}=\frac{1}{m}\delta^{ab}\left[{\bar{\partial_{a}\varphi }}_{x}-\beta A_{bx}\right],$$

is the drift velocity, and the spatial fluctuations $\Delta w^{a}$ obey

(43) $${\langle \Delta w^{a}\rangle }_{x}=0\;\mathrm{and}\;{\langle \Delta w^{a}\Delta w^{b}\rangle }_{x}=\frac{\eta }{m}\delta^{ab}\Delta t.$$

We are now ready to investigate the consequences of the spatial transition probability,

(44) $$P\left(x^{\prime }|x\right)=\frac{1}{Z}\exp\left[-\frac{m}{2\eta \Delta t}\;\delta_{ab}\left(\Delta x^{a}-\Delta {\bar{x}}_{x}^{a}\right)\left(\Delta x^{b}-\Delta {\bar{x}}_{x}^{b}\right)\right],$$

by rewriting the equation of evolution for the spatial distribution ρ(x), Equation (22), as a differential equation.

## 6. The Probability Evolution Equation

Multiply Equation (22) by a smooth test function $f_{x^{\prime }}$ and integrate over $x^{\prime }$,

(45) $$\int dx^{\prime }\;\rho_{x^{\prime }}^{\prime }f_{x^{\prime }}=\int dx^{\prime }\int dx\;P\left(x^{\prime }|x\right)\rho_{x}f_{x^{\prime }}=\int dx\left[\int dx^{\prime }P\left(x^{\prime }|x\right)\;f_{x^{\prime }}\right]\rho_{x}.$$

The test function $f_{x^{\prime }}$ is assumed sufficiently smooth precisely so that it can be expanded about *x*. Terms ${\left(\Delta x\right)}^{2}$ contribute to O(Δt). Then, dropping all terms of order higher than Δt, the integral in the brackets is

(46) $$\begin{array}{cc} \left[\cdots \right] & =\int dx^{\prime }\;P\left(x^{\prime }|x\right)\left(f_{x}+\frac{\partial f_{x}}{\partial x^{a}}\Delta x^{a}+\frac{1}{2}\frac{\partial^{2}f_{x}}{\partial x^{a}\partial x^{b}}\Delta x^{a}\Delta x^{b}+\ldots \right)\\ \; & =f_{x}+b_{x}^{a}\Delta t\frac{\partial f}{\partial x^{a}}+\frac{1}{2}\Delta t\;\frac{\eta }{m}\delta^{ab}\frac{\partial^{2}f}{\partial x^{a}\partial x^{b}}+\ldots \end{array}$$

where we used Equations (42) and (43). Dropping the primes on the left-hand side of (45), substituting (46) into the right, and dividing by Δt, gives

(47) $$\int dx\;\frac{1}{\Delta t}\left[\rho_{x}^{\prime }-\rho_{x}\right]f_{x}=\int dx\left[b_{x}^{a}\frac{\partial f_{x}}{\partial x^{a}}+\frac{1}{2}\;\frac{\eta }{m}\delta^{ab}\frac{\partial^{2}f_{x}}{\partial x^{a}\partial x^{b}}\right]\rho_{x}.$$

Next, integrate by parts on the right and let Δt→0. Since the test function $f_{x}$ is arbitrary, we conclude that

(48) $$\partial_{t}\rho =-\partial_{a}\left(b^{a}\rho \right)+\frac{1}{2}\frac{\eta }{m}\delta^{ab}\partial_{a}\partial_{b}\rho .$$

This can be written in the alternative form,

(49) $$\partial_{t}\rho_{t}=-\partial_{a}\left(v_{x}^{a}\rho_{x}\right),$$

which is a continuity equation, where

(50) $$v_{x}^{a}=b_{x}^{a}-\frac{\eta }{2m}\delta^{ab}\partial_{b}\log\rho_{x}$$

is the “current” velocity—the velocity of the probability current.

## 7. The e-Hamiltonian and the Pauli Equation

The whole purpose of this exercise has been to formulate the physical fact that particle paths are continuous in a way that allows us to identify a suitable notion of time. It is natural to require that acceptable Hamiltonians be such that they generate evolution along a time defined in terms of the very same “clock” (the system itself) that provides the measure of time. Therefore, our immediate goal is to identify those Hamiltonians $\tilde{H}$ that reproduce the continuity Equation (49). On the other hand, using Equations (1) and (3) we can write $\partial \rho_{x}/\partial t$ directly in Hamiltonian form. Since $\left(\rho_{kx},\xi_{kx}\right)$ are canonically conjugate, then

(51) $$\rho_{x}=\rho_{+x}+\rho_{-x}\;\mathrm{and}\;\xi_{x}=\frac{1}{2}\left(\xi_{+x}+\xi_{-x}\right)$$

are canonically conjugate too (we can check that $\left\{\rho_{x},\xi_{x^{\prime }}\right\}=\delta_{xx^{\prime }}$) which leads to

(52) $$\frac{\partial \rho_{x}}{\partial t}=\frac{\partial \rho_{+x}}{\partial t}+\frac{\partial \rho_{-x}}{\partial t}=\frac{\delta \tilde{H}}{\delta \xi_{+x}}+\frac{\delta \tilde{H}}{\delta \xi_{-x}}=\frac{\delta \tilde{H}}{\delta \xi_{x}}.$$

As we see from Equations (49) and (52), at this point in our argument, we have two independent expressions for $\partial \rho_{x}/\partial t$ and, therefore, there must exist some intimate connection between the drift potentials $\varphi_{kx}$ and the phases $\xi_{kx}$. To find what this relation might be, examine Equations (42) and (50) and rewrite the current velocity as

$$v_{x}^{a}=\frac{1}{m}\delta^{ab}\partial_{b}\sum_{k}\rho_{k|x}\left(\varphi_{kx}-\frac{\eta }{2}\log\rho_{x}\right)-\frac{\beta }{m}A_{x}^{a}.$$

We propose that the desired relation is

(53) $$\xi_{kx}=\varphi_{kx}-\eta \log\rho_{x}^{1/2},$$

so that

(54) $$v_{x}^{a}=\frac{\delta^{ab}}{m}\left(\sum_{k}\rho_{k|x}\partial_{b}\xi_{kx}-\beta A_{bx}\right).$$

We note that Equation (53) is a simple canonical transformation since $\varphi_{kx}$ and $\xi_{kx}$ differ by a function of the generalized coordinate $\rho_{kx}$ they are equally legitimate choices of canonical momenta—the choice of $\xi_{kx}$ over $\varphi_{kx}$ is purely a matter of convenience.

The other piece of information guiding our choice of e-Hamiltonian is the fact that for HK flows, the e-Hamiltonian is bilinear in the wave functions, Equation (16). The next step, therefore, is to write Equations (49) and (54) in terms of the wave functions, $\psi_{kx}=\rho_{kx}^{1/2}\exp\frac{i}{\hbar }\xi_{kx}$. After a little algebra, we find

(55) $$\frac{\partial \rho_{x}}{\partial t}=\frac{\hbar i}{2m}\sum_{k}\partial_{a}\left[\psi_{k}^{*}\left(D^{a}\psi_{k}\right)-\psi_{k}{\left(D^{a}\psi_{k}\right)}^{*}\right],$$

where $D_{a}=\partial_{a}-\frac{iq}{\hbar c}A_{a}$ is the covariant derivative. Further rearranging yields

(56) $$\frac{\partial \rho_{x}}{\partial t}=\frac{\hbar }{2mi}\left[\psi_{k}{\left(D_{a}D_{a}\psi_{k}\right)}^{*}-\psi_{k}^{*}D_{a}D_{a}\psi_{k}\right]=\frac{\delta \tilde{H}}{\delta \xi_{x}},$$

which is a *linear* functional differential equation for $\tilde{H}$ that is easily integrated,

(57) $$\tilde{H}\left[\psi ,\psi^{*}\right]=\frac{-\hbar^{2}}{2m}\int dx\;\;\sum_{k}\psi_{kx}^{*}D_{a}D_{a}\psi_{kx}+\tilde{V},$$

where the integration constant $\tilde{V}$ is independent of $\xi_{x}$. This is easily checked: let $\xi_{x}\to \xi_{x}+\delta \xi_{x}$ and use

(58) $$\delta \psi_{kx}=\frac{i}{\hbar }\psi_{kx}\;\delta \xi_{x}\;\mathrm{and}\;\delta \psi_{kx}^{*}=-\frac{i}{\hbar }\psi_{kx}^{*}\;\delta \xi_{x},$$

to get

(59) $$\delta \tilde{H}=\frac{\hbar }{2mi}\int dx\;\;\sum_{k}\left[\psi_{kx}{\left(D_{a}D_{a}\psi_{kx}\right)}^{*}-\psi_{kx}^{*}D_{a}D_{a}\psi_{kx}\right]\delta \xi_{x}\;\mathrm{qed}.$$

Thus, we see that the kinetic energy in the e-Hamiltonian (57) is traced to the entropic updating that led to the spatial continuity equation, while the potential energy is introduced as an integration constant. To determine $\tilde{V}$ we note that in order for $\tilde{H}$ to generate an HK flow we must require that $\tilde{V}$ be bilinear in ψ,

(60) $$\tilde{V}\left[\psi ,\psi^{*}\right]=\int dxdx^{\prime }\sum_{kk^{\prime }}\psi_{kx}^{*}{\hat{V}}_{kx,k^{\prime }x^{\prime }}\psi_{k^{\prime }x^{\prime }}$$

for some Hermitian kernel ${\hat{V}}_{kx,k^{\prime }x^{\prime }}$ and, furthermore, to reproduce the ED evolution in Equation (56), $\tilde{V}$ must be independent of $\xi_{x}=\left(\xi_{+x}+\xi_{-x}\right)/2$,

(61) $$\frac{\delta \tilde{V}}{\delta \xi_{x}}=\frac{\delta }{\delta \xi_{x}}\int dxdx^{\prime }\sum_{kk^{\prime }}\rho_{kx}^{1/2}\rho_{k^{\prime }x^{\prime }}^{1/2}{\hat{V}}_{kx,k^{\prime }x^{\prime }}\exp\frac{i}{\hbar }\left(\xi_{k^{\prime }x^{\prime }}-\xi_{kx}\right)=0.$$

To satisfy (61) for *arbitrary* choices of $\rho_{kx}$ and $\rho_{k^{\prime }x^{\prime }}$ we require that the kernel ${\hat{V}}_{kx,k^{\prime }x^{\prime }}$ be *local* in *x*,

(62) $${\hat{V}}_{kx,k^{\prime }x^{\prime }}=\delta_{xx^{\prime }}{\hat{V}}_{kk^{\prime }}\left(x\right),$$

The local Hermitian kernel ${\hat{V}}_{kk^{\prime }}\left(x\right)$ is a Hermitian 2×2 matrix which can be expanded in terms of Pauli matrices,

(63) $${\hat{V}}_{kk^{\prime }}\left(x\right)=V_{0}\left(x\right)\delta_{kk^{\prime }}+V_{a}\left(x\right)\sigma_{kk^{\prime }}^{a},\;\left(a=1,2,3\right),$$

where $V_{0}\left(x\right)$ and $V_{a}\left(x\right)$ are four scalar functions,

(64) $$\tilde{V}\left[\psi ,\psi^{*}\right]=\int dx\left(V_{0x}\sum_{k}\psi_{kx}^{*}\psi_{kx}+V_{ax}\sum_{kk^{\prime }}\psi_{kx}^{*}\sigma_{kk^{\prime }}^{a}\psi_{k^{\prime }x}\right)$$

(and we can easily check that, indeed, $\delta \tilde{V}/\delta \xi_{x}=0$). The final expression for the e-Hamiltonian

(65) $$\tilde{H}=\int dx\;\sum_{kk^{\prime }}\;\psi_{kx}^{*}\left[\left(\frac{-\hbar^{2}}{2m}D_{a}D_{a}+V_{0}\right)\delta_{kk^{\prime }}+V_{a}\sigma_{kk^{\prime }}^{a}\right]\psi_{k^{\prime }x},$$

takes the form of an expected value. The corresponding Schrödinger equation—the Pauli equation—is

(66) $$i\hbar \frac{\partial \psi_{kx}}{\partial t}=\frac{\delta \tilde{H}}{\delta \psi_{kx}^{*}}=\frac{-\hbar^{2}}{2m}\delta^{ab}D_{a}D_{b}\psi_{kx}+V_{0x}\psi_{kx}+V_{ax}\sigma_{kk^{\prime }}^{a}\psi_{k^{\prime }x}.$$

Incidentally, at this stage in the development, we see that the constants *ℏ*, *m*, and q/c can be safely given their usual meanings and that the entropic time *t* is the actual time measured by clocks—after all, it is using equations such as the Schrödinger or the Pauli equations that we calibrate our measuring devices.

For a *point* particle, such as an electron, the Pauli equation takes its simplest form,

(67) $$i\hbar \partial_{t}\Psi_{x}=\frac{1}{2m}{\left(\frac{\hbar }{i}\vec{\partial }-\frac{q}{c}\vec{A}\right)}^{2}\Psi_{x}+qA_{0}\Psi_{x}-\frac{\hbar q}{2mc}B_{a}\sigma^{a}\Psi_{x},$$

or

(68) $$i\hbar \partial_{t}\Psi_{x}=\frac{1}{2m}{\left[{\hat{\sigma }}^{a}\left(\frac{\hbar }{i}\partial_{a}-\frac{q}{c}A_{a}\right)\right]}^{2}\Psi_{x}+qA_{0}\Psi_{x},$$

where $\Psi_{x}$ is given in Equation (2) and

(69) $$V_{0x}=qA_{0}\left(x\right)\;\mathrm{and}\;V_{ax}=-\frac{\hbar q}{2mc}B_{a}\left(x\right)$$

represent the interactions of the charge with the scalar electric potential $A_{0}\left(x\right)$ and of the magnetic dipole with the magnetic field $B_{a}$. Equation (66) is more general in that it could describe extended particles such as protons and neutrons with anomalous magnetic moments or even particles with electric dipole moments. (The latter would violate time-reversal invariance and have not been observed so far).

One may note that Hilbert spaces have not been mentioned; strictly, they are not needed. However, the linearity of Equation (66) suggests that a Hilbert space is a convenient calculational tool [3,4]. Here, for completeness, we briefly mention how to introduce vectors |Ψ〉 in a linear Hilbert space. The map to the Dirac notation, $\psi_{kx}↔|\Psi \rangle$, is defined by

(70) $$|\Psi \rangle =\int dx\sum_{k}\;|kx\rangle \psi_{kx}\;\mathrm{where}\;\psi_{kx}=\langle kx|\Psi \rangle ,$$

where, in this “kx” representation, the vectors {|kx〉} form a basis that is orthogonal and complete,

(71) $$\langle kx|k^{\prime }x^{\prime }\rangle =\delta_{kk^{\prime }}\delta_{xx^{\prime }}\;\mathrm{and}\;\int dx\sum_{k}\;|kx\rangle \langle kx|=\hat{1}.$$

The Hilbert scalar product $\langle \Psi_{1}|\Psi_{2}\rangle$ is then defined by exploiting the structures already available to us, Equations (4), (6) and (12) for $\Psi^{\mu x}$, the symplectic form Ω and the metric *G*,

(72) $$\langle \Psi_{1}|\Psi_{2}\rangle \overset{\mathrm{def}}{=}\frac{1}{2\hbar }\int dxdx^{\prime }\sum_{\mu \mu^{\prime }}\left(G_{\mu x,\mu^{\prime }x^{\prime }}+i\Omega_{\mu x,\mu^{\prime }x^{\prime }}\right)\Psi_{1}^{\mu x}\Psi_{2}^{\mu^{\prime }x^{\prime }}.$$

A straightforward calculation yields the familiar expression

(73) $$\langle \Psi_{1}|\Psi_{2}\rangle =\int dx\;\sum_{k}\psi_{1kx}^{*}\psi_{2kx}.$$

The e-Hamiltonian is given by the expected value,

(74) $$\tilde{H}=\langle \Psi |\hat{H}|\Psi \rangle \;\mathrm{with}\;{\hat{H}}_{kx,k^{\prime }x^{\prime }}=\langle kx|\hat{H}|k^{\prime }x^{\prime }\rangle ,$$

where the matrix element ${\hat{H}}_{kx,k^{\prime }x^{\prime }}$ can be read off Equation (65).

## 8. Orbital and Spin Angular Momentum

We finally come to the “reconstruction” of spin. The central input is that angular momentum is the generator of rotations. Under a rotation $\vec{\zeta }=\zeta \vec{n}$ by an infinitesimal angle ζ about the axis $\vec{n}$ we have,

(75) $$\vec{x}\to {\vec{x}}_{\zeta }=\vec{x}+\zeta \vec{n}\times \vec{x}.$$

The action of rotations on the *spatial* probability distribution ρ(x) is given by

(76) $$\rho \left(x\right)\to \rho_{\zeta }\left(x\right)\;\mathrm{with}\;\rho_{\zeta }\left(x_{\zeta }\right)=\rho \left(x\right)\;\mathrm{or}\;\rho_{\zeta }\left(x\right)=\rho \left(x_{-\zeta }\right).$$

so that

(77) $$\delta_{\zeta }\rho \left(x\right)=\rho_{\zeta }\left(x\right)-\rho \left(x\right)=-\zeta \vec{n}\cdot \vec{x}\times \vec{\partial }\rho_{x}=-\varepsilon^{abc}\zeta n_{a}x_{c}\partial_{c}\rho_{x}.$$

Therefore

(78) $$\frac{\partial \rho_{x}}{\partial \zeta }=-\varepsilon^{abc}n_{a}x_{b}\partial_{c}\rho_{x}.$$

On the other hand, since rotations about $n^{a}$ are generated by the Hamiltonian function ${\tilde{J}}^{a}n_{a}$, and as we saw in Equation (51) $\left\{\rho_{x},\xi_{x}\right\}$ are canonically conjugate, we can write the Hamilton equation

(79) $$\frac{\partial \rho_{x}}{\partial \zeta }=\frac{\partial \rho_{+x}}{\partial t}+\frac{\partial \rho_{-x}}{\partial t}=\frac{\delta {\tilde{J}}^{a}}{\delta \xi_{+x}}n_{a}+\frac{\delta {\tilde{J}}^{a}}{\delta \xi_{-x}}n_{a}=\frac{\delta {\tilde{J}}^{a}}{\delta \xi_{x}}n_{a}\;.$$

Combining with (78) leads to

(80) $$-\varepsilon^{abc}n_{a}x_{c}\partial_{c}\rho_{x}=\frac{\delta {\tilde{J}}^{a}}{\delta \xi_{x}}n_{a},$$

which is a linear differential equation for ${\tilde{J}}^{a}n_{a}$. The integration is easy and since (80) holds for any choice of $n^{a}$, we find

(81) $${\tilde{J}}^{a}=\int dx\;\;\sum_{k}\psi_{kx}^{*}\left(\varepsilon^{abc}x_{b}\frac{\hbar }{i}\partial_{c}\right)\psi_{kx}+{\tilde{S}}^{a}.$$

The first integral is recognized as orbital angular momentum, and ${\tilde{S}}^{a}$ is an integration constant independent of $\xi_{x}$, which we identify as the *spin functional*. (Equation (81) is easy to check: just let $\xi_{x}\to \xi_{x}+\delta \xi_{x}$ with $\delta \psi_{kx}=i\psi_{kx}\;\delta \xi_{x}/\hbar$, then integrate by parts using $\varepsilon^{abc}\partial_{c}x^{b}=0$).

To determine the spin functional ${\tilde{S}}^{a}$, we impose two natural conditions: The first, mentioned in Section 3, is that ${\tilde{J}}^{a}n_{a}$ is a Hamiltonian function—it generates an HK flow. The second condition is motivated by choice of ontology: the two-valuedness of *k* requires that the action of ${\tilde{S}}^{a}$ on the wave functions given by Equation (2) results in a *2-dimensional* representation of the rotation group.

Concerning the first condition: in order for ${\tilde{J}}^{a}$ in (81) to generate an HK flow we require that ${\tilde{S}}^{a}$ be also bilinear in ψ,

(82) $${\tilde{S}}^{a}\left[\psi ,\psi^{*}\right]=\int dxdx^{\prime }\sum_{kk^{\prime }}\psi_{kx}^{*}{\hat{S}}_{kx,k^{\prime }x^{\prime }}^{a}\psi_{k^{\prime }x^{\prime }},$$

for some Hermitian kernel ${\hat{S}}_{kx,k^{\prime }x^{\prime }}^{a}$. Furthermore, to reproduce Equation (80), ${\hat{S}}_{kx,k^{\prime }x^{\prime }}^{a}$ must be independent of $\xi_{x}=\left(\xi_{+x}+\xi_{-x}\right)/2$,

(83) $$0=\frac{\delta {\tilde{S}}^{a}}{\delta \xi_{x}}=\frac{\delta }{\delta \xi_{x}}\int dxdx^{\prime }\sum_{kk^{\prime }}\rho_{kx}^{1/2}\rho_{k^{\prime }x^{\prime }}^{1/2}{\hat{S}}_{kx,k^{\prime }x^{\prime }}^{a}\exp\frac{i}{\hbar }\left(\xi_{k^{\prime }x^{\prime }}-\xi_{kx}\right),$$

for *arbitrary* choices of $\rho_{kx}$ and $\rho_{k^{\prime }x^{\prime }}$. It follows that ${\hat{S}}_{kx,k^{\prime }x^{\prime }}^{a}$ must be local in *x*,

(84) $${\hat{S}}_{kx,k^{\prime }x^{\prime }}^{a}=\delta_{xx^{\prime }}{\hat{S}}_{kk^{\prime }}^{a}\left(x\right)\;\mathrm{or}\;{\tilde{S}}^{a}=\int dx\sum_{kk^{\prime }}\psi_{kx}^{*}{\hat{S}}_{kk^{\prime }}^{a}\left(x\right)\psi_{k^{\prime }x}.$$

To implement the second condition, we recall that a 2-dimensional representation of rotations in terms of Cayley-Klein parameters was already known before the discovery of QM (see, e.g., [27]). In a 2×2 matrix representation a generic vector $\vec{x}$ is represented by

(85) $$\vec{x}=x^{a}\sigma_{a}=\left[\begin{array}{cc} x^{3} & x^{1}-ix^{2}\\ x^{1}+ix^{2} & -x^{3} \end{array}\right],$$

where the basis vectors ${\vec{e}}_{a}$ are represented by Pauli matrices $\sigma_{a}$, and a rotation by ζ about the axis $\vec{n}$ is represented by

(86) $${\vec{x}}^{\prime }={\hat{U}}_{\zeta }\vec{x}{\hat{U}}_{\zeta }^{-1}\;\mathrm{where}\;{\hat{U}}_{\zeta }=\exp\left(-in^{a}\sigma_{a}\zeta /2\right).$$

So far, this has nothing to do with QM, but the 2×2 matrices ${\hat{U}}_{\zeta }$ form a group and the corresponding rotation of the wave function Ψ is given by

(87) $$\Psi \left(x\right)\to \Psi_{\zeta }\left(x_{\zeta }\right)={\hat{U}}_{\zeta }\Psi \left(x\right)\;\mathrm{or}\;\Psi_{\zeta }\left(x\right)={\hat{U}}_{\zeta }\Psi \left(x_{-\zeta }\right),$$

which suggests we rewrite

(88) $${\hat{U}}_{\zeta }=\exp\left(-i{\hat{S}}^{a}\zeta /\hbar \right)\;\mathrm{with}\;{\hat{S}}_{kk^{\prime }}^{a}=\frac{\hbar }{2}\sigma_{kk^{\prime }}^{a}.$$

From (84) the spin functional we seek is

(89) $${\tilde{S}}^{a}\left[\psi ,\psi^{*}\right]=\int dx\sum_{kk^{\prime }}\psi_{kx}^{*}\frac{\hbar }{2}\sigma_{kk^{\prime }}^{a}\psi_{k^{\prime }x}.$$

The translation to Hilbert space in Dirac notation is, once again, straightforward. For example, the spin functional is the expected value,

(90) $${\tilde{S}}^{a}=\langle \Psi |{\hat{S}}^{a}|\Psi \rangle \;\mathrm{with}\;{\hat{S}}_{kx,k^{\prime }x^{\prime }}^{a}=\langle kx|{\hat{S}}^{a}|k^{\prime }x^{\prime }\rangle ,$$

where the matrix element ${\hat{S}}_{kx,k^{\prime }x^{\prime }}^{a}$ can be read off Equation (89). This concludes the reconstruction of spin.

## 9. Discussion and Conclusions

We have reconstructed the mathematical formalism for the QM of a spin-1/2 particle and recovered the linearity of the Pauli equation, the emergence of complex numbers, the peculiar properties of spin 1/2, and more. This guarantees that the ED predictions are in complete agreement with experiments, which is a feature that ED shares with so many other interpretations of QM. Where ED claims an additional dose of success is in achieving ontological and epistemic clarity. On the ontological side, it provides a crisp answer to the question “what is real?”: position and “*k*-ness” are real. ED affords clear epistemic status to probabilities, wave functions, energies, and angular momenta, including spin, and does this while enlisting the proven methods of modern quantitative epistemology, namely entropic methods and information geometry, without the need to invoke exotic or quantum probabilities. In this latter sense, ED is a rather conservative model. However, ED turns out to be radically non-classical in that it is a dynamics of probabilities and not of particles. ED denies the ontic status of dynamics, and it is the latter aspect of ED that violates our classical intuitions. To be clear, if at one instant, probabilities are large in one place, and at a later instant, they are large somewhere else, we are correct to believe that the particles have moved—but nothing in ED describes the causal mechanism that pushed the particles around. As far as we can tell, there is none; ED is a *mechanics without a mechanism*.

Now that the dynamics have been fully developed, we can revisit some questions raised back in Section 1. What is this mysterious, not classically describable *k*? What is spin? How are *k* and spin-related? What, if anything, is spinning?

Not much can be said about *k* except that it takes two values and it belongs in the ontic sector. Perhaps this is just the way the world is, and it is all one can ever hope to say. Concerning spin, much has been said in the previous section: it belongs in the epistemic sector, it generates HK flows and rotations, and much of its spinor structure follows from the two-valuedness of *k*.

The relation between *k* and spin is not without interest. The important difference is that the former is ontic and the latter epistemic—as different as different can be. However, there tends to exist a 1-1 correspondence between the ontic microstates (k=+1 and k=−1) and the epistemic states that represent certainty about them (the spinors 10 and 01) and this might be a source of confusion. However, some mystery is bound to remain. Consider a system in the epistemic state described by 10; we are certain that k=+1. Suppose the system is then rotated by θ=π/2 about the ${\vec{e}}_{y}$ axis,

(91) exp−iσyπ/2210=1211.

The outcome of this physical operation is that now we ought to believe that there is a 50% probability that a transition from k=1 to k=−1 has occurred. ED is silent about what could have caused this transition to happen. The weirdness of a *mechanics without a mechanism* can manifest itself not just in dynamics but also in the context of rotations or other operations.

Finally, there is the question of what is spinning? The assertions that probability, energy, momentum, and angular momentum, including spin, are not ontic but epistemic quantities force an extreme revision of our intuitions about physics. Probabilities may change, but they neither move nor flow; they are not substances. Similarly, accepting that spin is an epistemic concept forces us to revisit the intuition that something substantial is actually spinning. ED is just a model that describes a fictitious world. The real world out there may contain all sorts of things, but the fictitious world described in this paper contains a single point particle characterized by its position and its discrete *k* value, that is all. Within this ED model, the answer is clear: there is nothing there that could spin and, therefore, *nothing is spinning*.

The question, of course, is whether the ED models provide successful guidance in the real world and not just in their own fictitious worlds. So far, everything indicates they do.

## Data Availability

The original contributions presented in this study are included in the article. Further inquiries can be directed to the corresponding author.

## Abbreviations

The following abbreviations are used in this manuscript:

ED	Entropic Dynamics
QM	Quantum Mechanics
HK flows	Hamilton–Killing flows
